# Global health initiatives and mycetoma management: the unmet promise

**DOI:** 10.1093/trstmh/traf001

**Published:** 2025-02-04

**Authors:** Ahmed Hassan Fahal, Dallas J Smith, Ali Awadalla Saeed, Borna Nyaoke, Fabiana Alves, Kingsley Asiedu, Rod Hay

**Affiliations:** The Mycetoma Research Center, University of Khartoum, Khartoum 11111, Sudan; Mycotic Diseases Branch, U.S. Centers for Disease Control and Prevention, Atlanta, Georgia, USA; The Mycetoma Research Center, University of Khartoum, Khartoum 11111, Sudan; Faculty of Pharmacy, The National University, Khartoum, Sudan; Drugs for Neglected Diseases Initiative, Nairobi, Kenya; Drugs for Neglected Diseases Initiative, Geneva, Switzerland; World Health Organization, Geneva, Switzerland; King's College, London, UK

Global health initiatives have undeniably fuelled substantial progress in combating many neglected tropical diseases (NTDs). These efforts have been largely facilitated by international partnerships, collaborative funding and targeted programmes (frequently involving mass drug administration [MDA]) that have brought visibility and resources to diseases that are targeted for elimination like lymphatic filariasis, trachoma and onchocerciasis, with 49 countries having eliminated at least one NTD. Through programmes aligned with the WHO, governments, non-profit and pharmaceutical companies, millions have benefited from prevention and treatment efforts, underscoring the impact of focused health interventions. However, these success stories reveal critical disparities, for example, diseases and conditions amenable to case management such as lymphoedema and hydrocele from lymphatic filariasis and mycetoma. In this article, we focus on the challenges of mycetoma, a debilitating NTD that remains conspicuously underserved. The case of mycetoma underscores significant gaps in the failed commitments of global health and highlights how these initiatives have often fallen short of the stated goals to address all NTDs comprehensively within the framework of Universal Health Coverage and Sustainable Development.

Mycetoma, a complex and chronic infection that primarily affects marginalised populations in arid tropical and subtropical regions, manifests as progressive swelling and deformity, leading to severe disability if left untreated. The disease, which can be caused by either bacterial pathogens leading to actinomycetoma or fungi causing eumycetoma, is prevalent in countries like Sudan, Somalia, Senegal, Chad, India and Mexico. Its impact is particularly devastating in rural areas, where lack of awareness and limited access to healthcare facilities mean that the disease is frequently diagnosed late, by which stage extensive mutilating surgical intervention and amputation are often the only options. Prevention of mycetoma is difficult, and no environmental interventions to decrease the fungal or bacterial load in the environment have been studied. The physical and socioeconomic toll of mycetoma is profound, affecting the individual's ability to work, attend school and participate in daily life, leading to isolation, mental health disability, stigmatisation, social exclusion and a cycle of illness and poverty. Despite these realities, mycetoma has historically received little attention, often because it lacks the scale of prevalence or the immediate fatality associated with other NTDs, in addition to the complexity of diagnosis and management, making it less attractive for funding and research.

The WHO-NTD Roadmap for 2021–2030 laid out the need to have mycetoma included in national control programmes and surveillance systems, and defined clear actions required for enhancing mycetoma management, identifying key areas for improvement such as early point-of-care diagnosis, accessible treatment and targeted research. However, progress on these crucial indicators has been limited, and the outlined goals remain largely unmet. In the 2024 WHO global report on NTDs, only seven countries reported mycetoma to the WHO, two countries included mycetoma in their national NTD action plans and there were no disability-adjusted life years estimates. Despite the roadmap recognition of mycetoma as a significant health concern, and mycetoma being classified a disease of high priority in the WHO fungal priority pathogen list, there has been little measurable advancement in increasing diagnostic capabilities, expanding treatment accessibility, or boosting research funding specific to mycetoma. This slow progress highlights persistent gaps in both research and resource allocation as well as implementation strategies, suggesting that further commitment and focused interventions are urgently needed if the WHO goals for mycetoma are to be realised.

Global health initiatives, despite their far-reaching impact on other diseases, have yet to offer mycetoma the resources needed for effective management. This oversight can be attributed to multiple factors, including the complexity of the disease itself, which requires specialised diagnostic techniques, and long, costly treatment regimens that are often inaccessible or unaffordable in low-resource settings. Unlike many NTDs that benefit from MDA programmes, mycetoma lacks a simple, scalable solution due to the extended treatment duration and the toxicity and expense of current medications. Because there is no early access to diagnosis, many cases present with advanced disease, demanding surgical interventions in most eumycetoma cases, which is particularly challenging in rural settings with limited medical infrastructure. These factors contribute to a vicious cycle of underdiagnosis, undertreatment and compound neglect.

Moreover, mycetoma remains underfunded in the research and development landscape. While billions of dollars have been mobilised globally for NTD research, the proportion allocated to mycetoma is minuscule. The 2023 G-finder report found that only US$0.5 million was given to mycetoma research in 2022, down by US$0.3 million from 2020 and 2021. Despite this neglect and limited investment, it is possible to perform research on mycetoma in endemic countries, if appropriate resources are made available. One notable achievement is the successful implementation of the first randomised clinical trial for eumycetoma, a collaboration between the Drugs for Neglected Diseases Initiative, the Mycetoma Research Center in Sudan and EISAI. This study showed promising results of an alternative therapy with fosravuconazole that would be more patient friendly, and the partners will continue to pursue its development to make it accessible to patients. Nevertheless, the lack of funding on more innovation for mycetoma has severely limited advances in diagnostics, treatments and public health strategies to combat the disease. Consequently, patients continue to rely on outdated, expensive medications with limited effectiveness, and there is little progress or support for developing more accessible and effective solutions. The absence of investment in mycetoma research further compounds the inequalities within global health funding structures, as the disease continues to persist in the poorest, most underserved regions, reinforcing the disparities it perpetuates.

The unmet promise of global health initiatives in addressing mycetoma points to the need for a renewed commitment from international health organisations, pharmaceutical companies, donors and governments to support a more inclusive and equitable approach to NTDs. An effective response to mycetoma requires targeted funding for research into affordable and accessible point-of-care diagnostic tools, as well as continued investment in new, less toxic and more effective therapies. This would allow for an integrated approach of early diagnosis and treatment at community level, decreasing morbidity and avoiding progression to severe disease, as well as promoting awareness campaigns and community mobilisation. Raising awareness about mycetoma among healthcare professionals and affected communities is essential to improving early diagnosis and treatment outcomes. Global health frameworks should more explicitly prioritise mycetoma within their agendas to ensure that it does not remain on the periphery of NTD action plans.

Addressing the gaps in mycetoma management requires a concerted, multistakeholder effort that recognises the disease's unique challenges and mobilises targeted actions to close these gaps effectively. Effective management, therefore, necessitates a unified response from healthcare providers, researchers, policymakers, non-governmental organisations and affected communities to address its complex, multifaceted challenges. In particular, incorporating mycetoma into skin-NTD initiatives can leverage resources and synergise programmatic and scientific work.

To improve mycetoma outcomes, focusing on and expanding research is critical. This includes exploring novel therapeutic approaches, understanding regional epidemiology and developing more accurate and affordable point-of-care diagnostics.

Equally important is enhancing access to effective treatments. Existing treatments for mycetoma are often lengthy and expensive, and they have limited availability in endemic regions. The cost burden falls directly on the patient. Itraconazole, often considered first-line therapy for eumycetoma, is frequently unavailable or unaffordable in many regions, and clinicians have to resort to using ketoconazole, an antifungal whose use has been discontinued in many countries because of the risk of adrenal and hepatotoxicity. Better access to treatment can be achieved by facilitating the supply and affordability of antifungal medications, exploring public–private partnerships to enhance drug distribution and promoting locally sustainable manufacturing processes. Pooled-procurement mechanisms for antifungals, ideally from regional manufacturers, can decrease costs and may encourage ministries of health to stock antifungals nationally. Integrating mycetoma into global health agendas would also signal a commitment to allocate resources and infrastructure for its management. This would involve including mycetoma within broader NTD frameworks, enabling the development of sustainable programmes dedicated to both prevention and care.

The case of mycetoma underscores a broader truth: the success of global health initiatives must be measured by their inclusivity and reach. While much global health funding targets high-profile diseases, such as HIV, TB and malaria, achieving true global health equity requires extending similar urgency and resources to all diseases that impact the lives of vulnerable populations. By prioritising mycetoma and other neglected conditions, we not only serve those who have been historically overlooked but also set a precedent for comprehensive healthcare systems that consider the needs of every patient.

The tools, research priority needs and knowledge to address mycetoma are available. What is lacking is the global will to act. By approaching neglected diseases like mycetoma with empathy, urgency and proactive measures, we can transform global health into a reality for all.

